# Impacts of Triglyceride Glucose-Waist to Height Ratio on Diabetes Incidence: A Secondary Analysis of A Population-Based Longitudinal Data

**DOI:** 10.3389/fendo.2022.949831

**Published:** 2022-07-22

**Authors:** Wenting Xuan, Dixing Liu, Jiana Zhong, Huijin Luo, Xiuwei Zhang

**Affiliations:** ^1^ Department of Endocrinology, Affiliated Dongguan Hospital, Southern Medical University (Dongguan People’s Hospital), Dongguan, China; ^2^ Department of Endocrinology, The Dongguan Affiliated Hospital of Jinan University, Binhaiwan Central Hospital of Dongguan, Dongguan, China

**Keywords:** type 2 diabetes mellitus, waist-to-height ratio, triglyceride−glucose index, TyG-WHtR, obesity, insulin resistance

## Abstract

**Background:**

The anthropometric indices (body mass index [BMI], waist circumference [WC] and waist-to-height ratio [WHtR]), triglyceride-glucose (TyG) index and TyG-related indicators (TyG-WHtR, TyG-BMI, TyG-WC) have been well documented to be highly correlated with insulin resistance (IR) and type 2 diabetes mellitus (T2DM). However, it was not immediately obvious which indicator would be optimal for screening people at risk of T2DM. Hence, this study intended to compare the predictive effects of the aforementioned markers on T2DM and to investigate the relation between baseline TyG-WHtR and incident T2DM.

**Methods:**

This longitudinal study included 15464 study population who were involved in the NAGALA (NAfld in the Gifu Area Longitudinal Analysis) study from 2004 to 2015. The TyG index was defined as ln [FPG (mg/dL) ×fasting TG (mg/dL)/2]. And the TyG-WHtR was calculated as TyG index ×WHtR. We divided the participants into four groups according to the TyG-WHtR quartiles. The primary endpoint was the incidence of diabetes.

**Results:**

After a median follow-up of 5.4 years, 2.4% (373/15464) participants developed diabetes. The incidence of diabetes increased with ascending TyG-WHtR quartiles (*P* for trend<0.001). Multivariable Cox proportional hazard analysis showed that a one-unit increase in TyG-WHtR was independently correlated with a 2.714-fold higher risk of diabetes [hazard ratio (HR) 2.714, 95% confidence interval (CI) 1.942-3.793; *P*<0.001). Stratification analysis revealed that increased TyG-WHtR (per 1-unit) was consistently correlated with diabetes incidence in different subgroups. Moreover, TyG-WHtR outperformed the other parameters by presenting the biggest area under the ROC curve (AUC) in men (AUC 0.746, 95% CI 0.716-0.776, *P*<0.001). However, all pairwise comparisons of AUC between TyG-WHtR and other indicators were not statistically different except TyG-WHtR vs. WHtR in women.

**Conclusions:**

A high TyG-WHtR is an important predictor of the increased cumulative risk of diabetes development. TyG-WHtR outperforms TyG, WHtR, TyG-WC and TyG-BMI in screening individuals who are susceptible to T2DM, especially in men.

## Background

The global prevalence of diabetes in 20-79 years old population has reached pandemic proportions, rising from an estimated prevalence of 10.5% (536.6million adults) in 2021 to 12.2% (783.2 million) in 2045 according to the 10th edition of IDF Diabetes Atlas Report (1). Type 2 diabetes mellitus (T2DM) has emerged as a common and serious chronic disease of our times, resulting in costly diabetes-related complications (2). Moreover, it is closely correlated with cardiovascular disease, malignancy, as well as increased all-cause mortality, reducing life expectancy and imposing an enormous economic burden on our society (3). Based on these facts, emphasizing the necessity to explore a convenient and effective method for early detection and prediction of diabetes is of paramount importance.

It is well known that insulin resistance (IR) plays a critical role in the pathogenesis of T2DM. Currently, the glucose clamp technique offers a highly accurate gold standard approach for diagnosing IR (4). Besides, HOMA-IR (homeostasis model assessment of IR), which was determined by fasting insulin level and blood glucose, is considered to be a commonly used measure to assess IR (5). However, glucose clamp is comparatively time-consuming, complicated to operate and expensive in real-world clinic. On the other hand, the measurement of insulin concentration is not routine in daily clinical practice. Thus, based on the limitations of both glucose clamp and HOMA-IR, it is relatively difficult to popularize in the epidemiologic investigations and screening individuals at risk of diabetes on a large-scale.

Triglyceride-glucose (TyG) index, a derivative of commonly used clinical indicators (fasting blood glucose and triglyceride), showed a remarkable relationship with glucose clamp and HOMA-IR (6, 7). In recent years, TyG index has been introduced as a reliable and promising surrogate marker for IR (8, 9). Unsurprisingly, numerous studies have shown that TyG index was closely relate to T2DM incidence (10–12). More recently, given the close association between obesity and IR, studies have also indicated that TyG-related indices comprising both TyG index and obesity-related anthropometric parameters (e.g., TyG-BMI, TyG-waist circumference [WC], TyG-waist to height ratio [WHtR]) may superior to TyG alone for predicting IR and cardiometabolic risk (13–15). Subsequent studies also highlighted that TyG-WHtR, TyG-BMI and TyG-WC were all associated with T2DM, respectively (16–18). However, there is still controversy about which of these markers can be established as the most effective and optimal parameter for screening individuals at risk of diabetes. For example, a cross-sectional study has shown that TyG index was superior to other TyG-derived parameters in identifying the occurrence of T2DM among the elderly (16). While Er LK et al. demonstrated that TyG-BMI exhibited a greater ability for the identification of T2DM than TyG alone (19). Furthermore, there is evidence showing that WHtR is superior to BMI or WC in predicting the incident diabetes (20–22). Thus, given these findings, it could be expected that TyG-WHtR may be a better predictor of T2DM than TyG-WC, TyG-BMI and even TyG alone. However, to our knowledge, no prior studies have ever been published yet comparing the predictive potential of TyG-WHtR and other TyG-related parameters for incident diabetes. Therefore, whether the TyG-WHtR has a better performance on T2DM prediction remains unclear.

Thus, this longitudinal study aimed to explore the relationship between the baseline TyG-WHtR and incident diabetes. And further compare the TyG-WHtR and other parameters (TyG, WHtR, TyG-WC and TyG-BMI) for predicting of T2DM in a large-scale Japanese cohort.

## Materials and methods

### Data Source

The data used in the present study were downloaded from Dryad Digital Repository (www.datadryad.org), which is freely available to other researchers. This original data has been initially analyzed and shared by Okamura T et al. in the https://doi.org/10.5061/dryad.8q0p192 (23). The following information was included in this open-access database: gender, age, weight, diastolic blood pressure (DBP), systolic blood pressure (SBP), WC, BMI, HbA1c, fasting plasma glucose (FPG) and lipid profile (triglyceride, HDL-C, total cholesterol). Besides, the database also contains alanine aminotransferase (ALT), gamma-glutamyl transferase (GGT), aspartate aminotransferase (AST), alcohol consumption, smoking status, exercise, fatty liver, follow-up duration and incident diabetes. Among them, the lifestyle habits of study population were based on a standardized questionnaire. The weekly alcohol consumption over the past month was categorized as none, minimal (<40g/week), light (40–140g/week), moderate (140–280g/week), and high consumption (>280g/week). As for smoking status, the study participants were classified as current, past or never smoker. And regular exercise was defined as taking part in any type of sports >1×/week regularly.

### Study Population

In this population-based longitudinal study, we selected the individual who joined the medical examination in Murakami Memorial Hospital (Gifu, Japan) between 2004 and 2015, and then performed a follow-up on incident diabetes. In total, 15464 participants (7034 women and 8430 men) were involved in the current study. The exclusion criteria for this clinical research were summarized as follows: 1) missing relevant data; 2) fatty liver disease, viral hepatitis B or C at baseline; 3) the habit of drinking heavily (alcohol consumption over 60g/d for males and 40g/d for females) at baseline; 4) any medication usage at baseline; 5) diagnosed with diabetes or FPG≥6.1 mmol/L at baseline. The ethics approval of the original study protocol was obtained from Murakami Memorial Hospital, and written informed consent was acquired from each patient for the collection and use of their data.

### Measurement of TyG and TyG-Related Parameters

The TyG index was defined as ln [FPG (mg/dL) ×fasting TG (mg/dL)/2] (6). And the formula of TyG-WHtR was as follows: TyG-WHtR=TyG index ×WHtR. Similarly, the TyG-WC and TyG-BMI were calculated as TyG index ×WC and TyG index ×BMI (17).

### Ascertainment of Diabetes

The incident diabetes of participants during follow-up was defined as FPG ≥7mmol/L, or HbA1c ≥6.5%, or patient self-report (24).

### Statistical Analysis

All analyses were performed with IBM SPSS 26.0 (Armonk, NY, USA), R software version 4.0.1 (R Foundation, Vienna, Austria) and Empower Stats (X&Y Solutions, Inc., Boston, MA). Continuous variables were expressed as mean ± standard deviation (SD) or median (25th and 75th percentiles). Categorical variables were shown as frequency (percentages). The enrolled patients were divided into four groups according to the TyG-WHtR quartiles. The statistical differences among the four groups were evaluated using One-way ANOVA for data with normal distribution and Kruskal-Wallis tests for non-normally distributed variables. The differences between categorical variables were compared using chi-square tests. The cumulative diabetes free rates according to the baseline TyG-WHtR quartiles were detected by Kaplan-Meier analysis, and the differences among groups were determined by log-rank tests. Then, we used Cox proportional hazards models to estimate the association between baseline TyG-WHtR and diabetes incidence after adjusting for confounders, including age, gender, BMI, SBP, DBP, TC, HDL-C, smoking status, alcohol consumption, exercise habit and fatty liver disease. In addition, a generalized additive model was also conducted to estimate the nonlinear relationship between TyG-WHtR and diabetes. To further verify the robustness of the results and find interactions, stratified analyses according to age (<40 and ≥40 years), sex, BMI (<24; ≥24, <28; and ≥28 kg/m^2^), SBP (<140 and ≥140 mmHg), DBP (<90 and ≥90 mmHg), TC (<5.2 and ≥5.2 mmol/L), HDL-C (normal HDL-C and low HDL-C), fatty liver disease, habit of exercise, smoking status (never, past and current), and alcohol consumption (non, light, moderate, and heavy) were implemented. And the likelihood ratio tests were conducted to evaluate the interactions among subgroups. The discriminative power of WHtR, TyG-related parameters and TyG for T2DM was evaluated by receiver operating characteristic (ROC) analysis. Then, the pairwise comparisons of areas under the receiver operating characteristic curves (AUCs) between TyG-WHtR and other parameters were applied. A *P*-value<0.05 (two-tailed) was considered statistically significant.

## Results

### Baseline Characteristics of Study Population

A total of 15464 people (mean age: 43.7 ± 8.9 years; 54.5% men and 45.5% women) were included in our study. The follow-up periods of these participants ranged from 0.5 years (164 days) to 13.0 years (4732 days). During the median 5.4-year follow-up duration, 373 (2.4%) participants developed diabetes. Baseline characteristics of study population stratified by TyG-WHtR quartiles were shown in **Table 1**. Patients in the highest TyG-WHtR quartile group (Q4) exhibited higher age, BMI, WC, WHtR, SBP, DBP and consisted of a greater proportion of men, drinkers, smokers, and fatty liver disease than the other groups (Q1-Q3). In terms of laboratory indicators, the patients with the higher TyG-WHtR values had higher levels of FBG, HbA1c, total cholesterol (TC), triglycerides, ALT, AST and GGT, but lower levels of HDL-C. Moreover, the levels of TyG index and other TyG-related parameters (TyG-BMI and TyG-WC) were all significantly higher in patients with high TyG-WHtR.

**Table 1:** Baseline characteristics of patients stratified by TyG-WHtR quartiles.

	TyG-WHtR quartiles				
Variables	Q1 (≤3.28) (n=3866)	Q2 (3.29-3.68) (n=3866)	Q3 (3.69-4.13) (n=3866)	Q4 (≥4.14) (n=3866)	*P* value
Age (years)	40.1 ± 8.1	43.0 ± 8.6	45.2 ± 8.7	46.6 ± 8.9	<0.001
Male (n, %)	1096 (28.3%)	1906 (49.3%)	2536 (65.6%)	2892 (74.8%)	<0.001
Follow up duration (days)	2041.0 (1045.3-3506.0)	1897.0 (916.8-3347.0)	2090.0 (1043.0-3483.0)	1888.0 (859.5-3374.3)	0.069
BMI (kg/m^2^)	19.2 ± 1.7	21.0 ± 1.7	22.7 ± 1.9	25.5 ± 2.9	<0.001
WC (cm)	66.8 ± 4.9	73.3 ± 4.9	79.0 ± 4.8	86.7 ± 6.8	<0.001
WHtR	0.41 ± 0.02	0.45 ± 0.02	0.48 ± 0.02	0.52 ± 0.04	<0.001
TyG	7.4 ± 0.4	7.8 ± 0.4	8.2 ± 0.4	8.7 ± 0.5	<0.001
TyG-BMI	141.7 ± 13.5	164.6 ± 12.8	185.8 ± 14.9	222.3 ± 26.2	<0.001
TyG-WC	493.1 ± 40.4	573.6 ± 36.2	646.9 ± 39.7	755.5 ± 68.2	<0.001
TyG-WHtR	3.0 ± 0.2	3.5 ± 0.1	3.9 ± 0.1	4.5 ± 0.4	<0.001
SBP (mmHg)	106.0 ± 12.3	111.5 ± 12.9	116.7 ± 13.6	123.7 ± 14.9	<0.001
DBP (mmHg)	65.7 ± 8.5	69.4 ± 9.2	73.1 ± 9.7	78.2 ± 10.2	<0.001
TC (mmol/L)	4.7 ± 0.8	5.0 ± 0.8	5.2 ± 0.8	5.5 ± 0.9	<0.001
Triglycerides (mmol/L)	0.4 (0.3-0.6)	0.6 (0.5-0.8)	0.9 (0.7-1.1)	1.4 (1.0-1.9)	<0.001
HDL-C (mmol/L)	1.7 (1.4-1.9)	1.5 (1.3-1.8)	1.3 (1.1-1.6)	1.2 (1.0-1.3)	<0.001
FPG (mmol/L)	4.9 ± 0.4	5.1 ± 0.4	5.2 ± 0.4	5.4 ± 0.4	<0.001
HbA1c (%)	5.1 ± 0.3	5.1 ± 0.3	5.2 ± 0.3	5.3 ± 0.3	<0.001
ALT (IU/L)	14 (11-17)	15 (12-19)	18 (14-24)	24 (17-33)	<0.001
AST (IU/L)	16 (13-19)	16 (13-20)	17 (14-21)	20 (16-24)	<0.001
GGT (IU/L)	12 (10-15)	14 (11-18)	17 (13-24)	23 (16-35)	<0.001
Fatty liver disease (n, %)					<0.001
Yes	18 (0.5%)	156 (4.0%)	625 (16.2%)	1942 (52.2%)	
No	3848 (99.5%)	3710 (96.0%)	3241 (83.8%)	1924 (49.8%)	
Alcohol consumption, g/week	1.0 (0.0-18.0)	1.0 (0.0-60.0)	4.2 (0.0-84.0)	12.0 (0.0-90.0)	<0.001
Smoking status (n, %)					<0.001
never	2893 (74.8%)	2412 (62.4%)	1998 (51.7%)	1728 (44.7%)	
past	432 (11.2%)	670 (17.3%)	881 (22.8%)	969 (25.1%)	
current	541 (14.0%)	784 (20.3%)	987 (25.5%)	1169 (30.2%)	
Habit of exercise (n, %)					<0.001
Yes	711(18.4%)	743 (19.2%)	685 (17.7%)	570 (14.7%)	
No	3155 (81.6%)	3123 (80.8%)	3181 (82.3%)	3296 (85.3%)	

BMI, body mass index; WC, waist circumference; WHtR, waist-to-height ratio; TyG: triglyceride-glucose; SBP, systolic blood pressure; DBP, diastolic blood pressure; HDL-C, high-density lipoprotein-cholesterol; TC, total cholesterol; FBG, fasting blood glucose; HbA1c, glycosylated haemoglobin A1c; ALT, alanine aminotransferase; AST, aspartate aminotransferase; GGT, gamma-glutamyl transferase.

### Clinical Outcome and Kaplan-Meier Analysis

**Figure 1:**
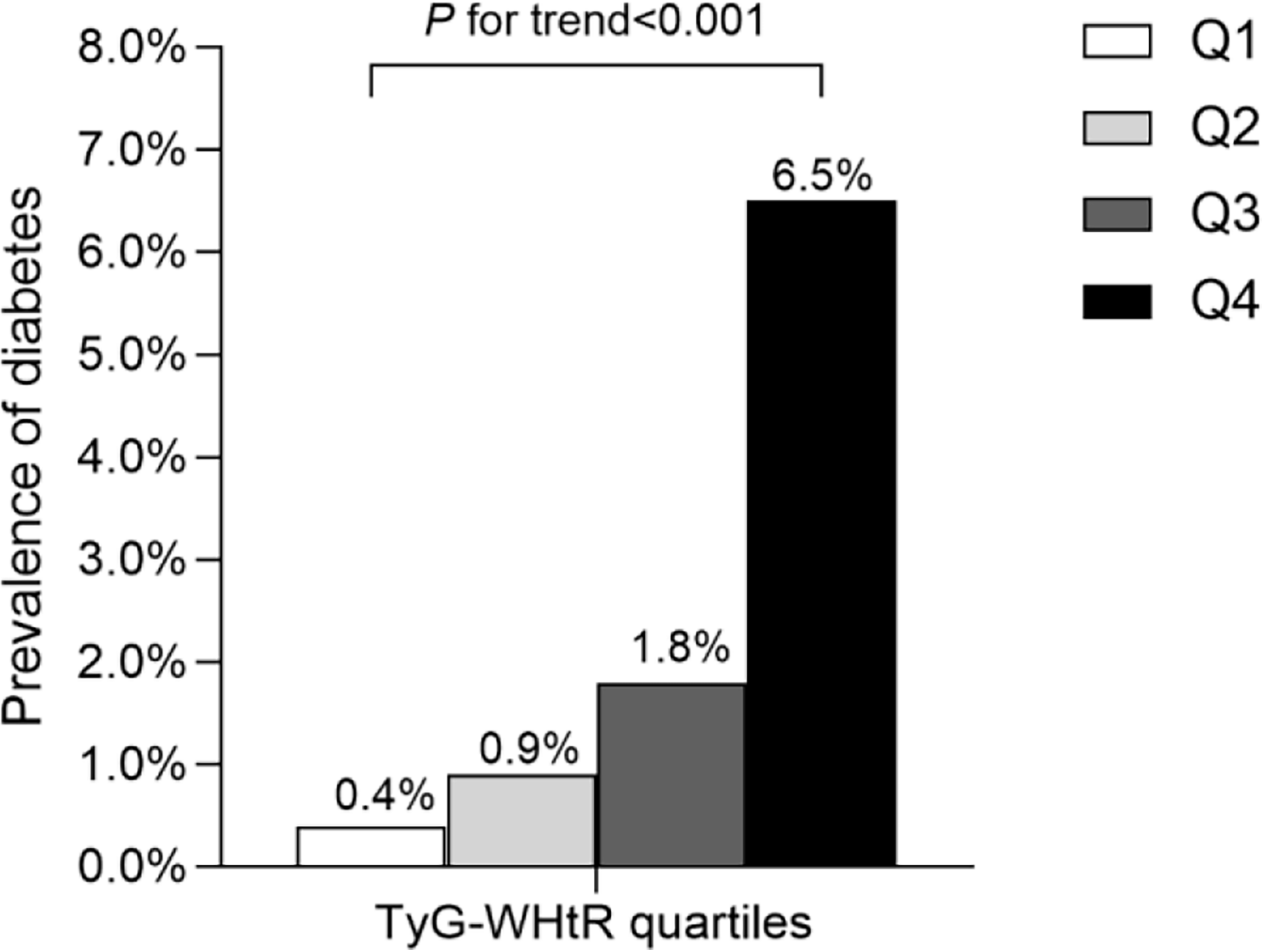
Prevalence of diabetes according to the baseline TyG-WHtR quartiles. All of the study participants were divided into four groups according to quartiles of TyG-WHtR (quartile 1 [Q1]: ≤3.28; quartile 2 [Q2]: 3.29-3.68; quartile 3 [Q3]: 3.69-4.13; quartile 4 [Q4]: ≥4.14), the prevalence of diabetes increased with ascending quartiles of TyG-WHtR (*P* for trend <0.05). TyG-WHtR: triglyceride glucose-waist to height ratio.

**Figure 2:**
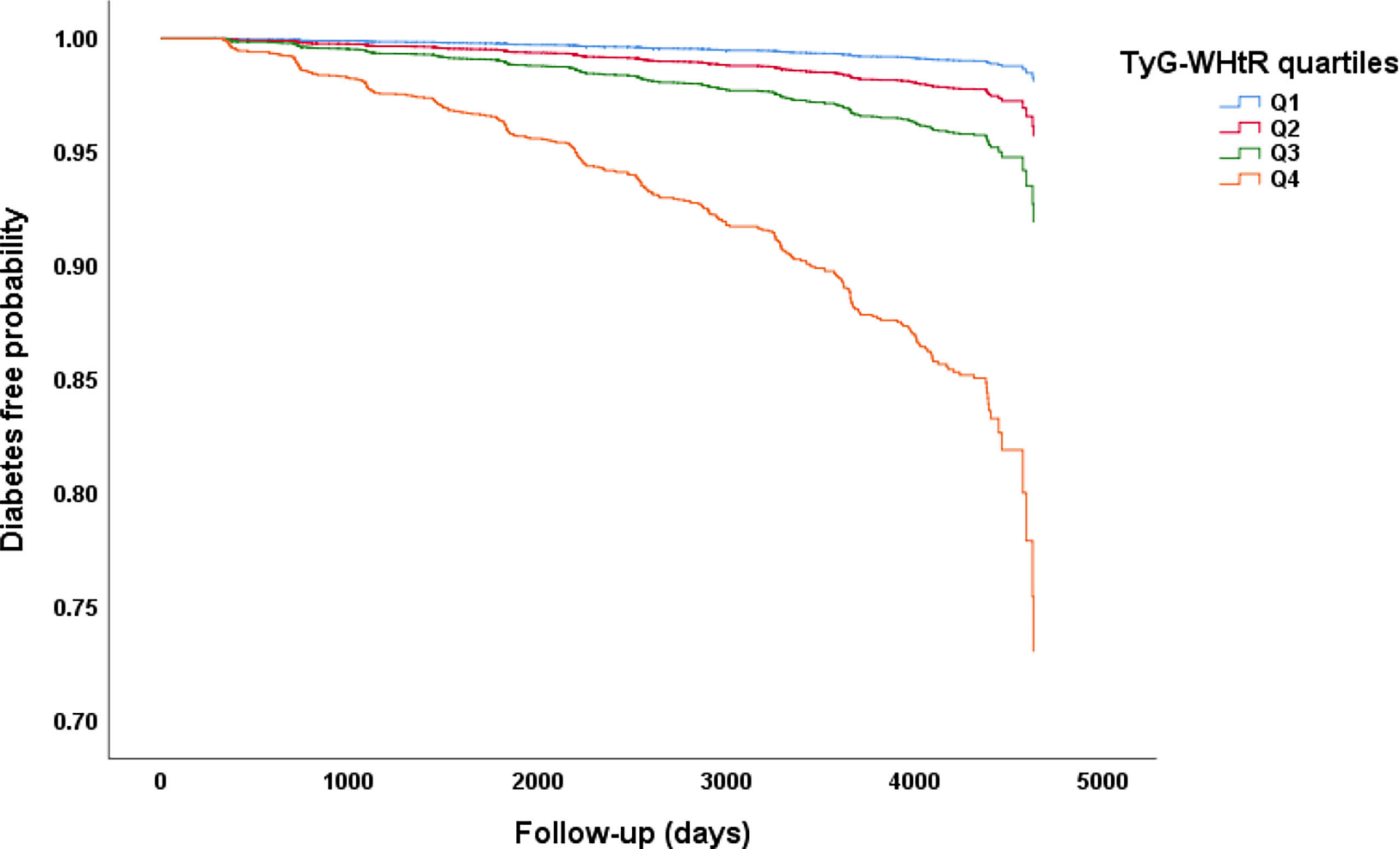
Kaplan–Meier curves of time to incident diabetes during follow-up. The patients were stratified by TyG-WHtR quartiles, the incidence of diabetes was significantly different among the TyG-WHtR quartile groups (Log-rank *P*<0.001). All pairwise comparisons were statistically significant (All *P*<0.05).

After a median follow-up of 5.4 years, 2.4% (373/15464) participants developed T2DM. As shown in **Figure 1**, we compared the incidence of diabetes among patients stratified by TyG-WHtR quartiles. Overall, the incidence of diabetes increased with ascending quartiles of TyG-WHtR (*P* for trend<0.001). The prevalence of T2DM was 0.4% in Q1, 0.9% in Q2, 1.8% in Q3, and 6.5% in Q4 group. The Kaplan-Meier curves of diabetes incidence grouped by TyG-WHtR quartiles were presented in **Figure 2**. The incidence of T2DM were significantly different among the TyG-WHtR quartile groups (Log-rank *P*<0.001). And all pairwise comparisons were also statistically significant (All *P*<0.05).

### Cox Regression Analysis to Evaluate the Relationship Between Baseline TyG-WHtR and Incident Diabetes

Multivariable Cox proportional hazard analysis was performed to analyze the independent effect of the baseline TyG-WHtR on the risk of developing diabetes (**Table 2**). After adjusting for age and gender, significant association existed between TyG-WHtR (as a continuous variable) and T2DM (HR 4.828, 95% CI 4.123-5.652; *P*<0.001). Further adjustment of BMI, SBP, DBP, TC and HDL-C, the higher TyG-WHtR was still independently related to the higher risk of diabetes (HR 3.457, 95% CI 2.504-4.774; *P*<0.001). Besides, the relationship remained significant after taking fatty liver disease, alcohol consumption, smoking status and exercise habit into account. In model 3, according to the result, a 1-unit increase in TyG-WHtR index was strongly correlated with a 2.714-fold greater risk of diabetes (HR 2.714, 95% CI 1.942-3.793; *P*<0.001).

**Table 2:** Multivariable Cox proportional hazards regression analysis of the association between baseline TyG-WHtR and incident diabetes.

	TyG-WHtR as a continuous variable		
	HR	95% CI	*P* value
Crude model	5.155	4.449-5.973	<0.001
Model 1	4.828	4.123-5.652	<0.001
Model 2	3.457	2.504-4.774	<0.001
Model 3	2.714	1.942-3.793	<0.001

Model 1: adjusted for age and gender.

Model 2: adjusted for age, gender, BMI, SBP, DBP, TC and HDL-C.

Model 3: adjusted for age, gender, BMI, SBP, DBP, TC, HDL-C, smoking status, alcohol consumption, exercise habit and fatty liver disease.

HR, hazard ratio; CI, confidence interval; TyG-WHtR, triglyceride glucose-waist to height ratio; BMI, body mass index; SBP, systolic blood pressure; DBP, diastolic blood pressure; TC, total cholesterol; HDL-C, high-density lipoprotein-cholesterol.

### Smoothing Function Analysis of TyG-WHtR on Incident Diabetes

**Figure 3:**
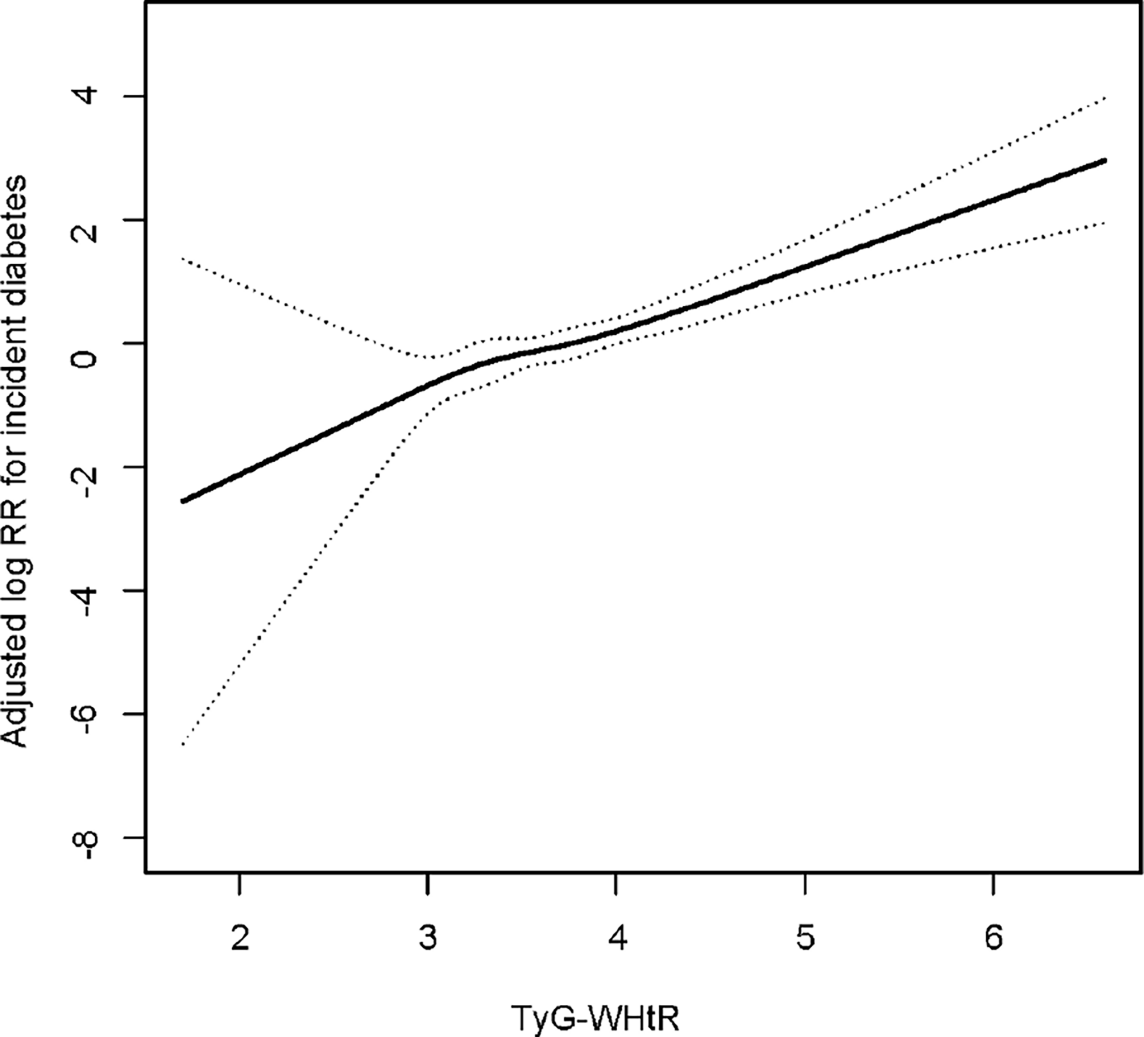
Dose–response relationship between TyG-WHtR and incident diabetes. Adjusted for age, gender, BMI, SBP, DBP, TC, HDL-C, smoking status, alcohol consumption, exercise habit and fatty liver disease.

The shape of the correlation between the baseline TyG-WHtR and the risk of diabetes was further conducted using a Cox proportional hazards regression model with cubic spline functions. Adjusted smoothed plots showed a linear relationship between the baseline TyG-WHtR and incident diabetes (**Figure 3**). The baseline TyG-WHtR magnitude was significantly positively associated with the log relative risk (log [RR]) for incident T2DM after adjusting for confounders, including age, gender, BMI, DBP, SBP, TC, HDL-C, alcohol consumption, smoking status, exercising habit and fatty liver disease.

### Stratification Analysis on the Association of TyG-WHtR with Incident Diabetes

**Figure 4:**
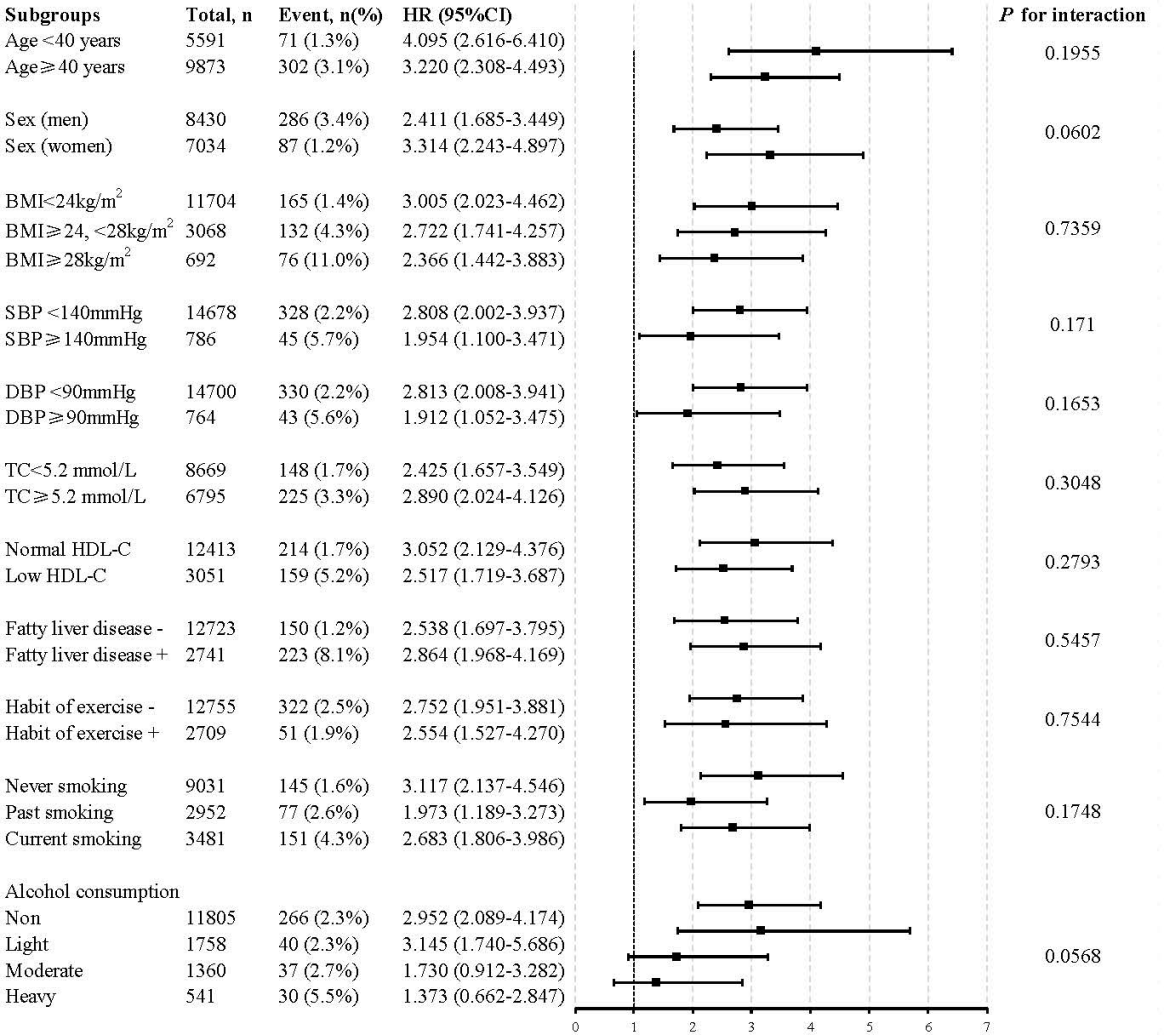
Stratification analysis on the association of the baseline TyG-WHtR with incident diabetes. HR was evaluated by 1−unit increase of TyG-WHtR. The model used in the stratified analyses consisted of all covariates used in Model 3 except for the variables that were used for stratification. HDL<1.04 mmol/L in men and <1.29 mmol/L in women was defined as low HDL-C. HR, hazard ratio; CI, confidence interval; TyG-WHtR, triglyceride glucose-waist to height ratio; BMI, body mass index; TC, total cholesterol; HDL-C, high-density lipoprotein-cholesterol.

Further evaluation of the risk stratification value of TyG-WHtR for diabetes incidence was performed in various subgroups. The model used in the stratified analyses consisted of all covariates used in Model 3 except for the variables that were used for stratification. The results showed that increased TyG-WHtR (per 1-unit) was consistently correlated with diabetes incidence in different subgroups, including age<40 or ≥40 years, men or women, SBP<140 or ≥140 mmHg, DBP<90 or ≥90 mmHg, BMI<24 or ≥24, <28 or ≥28 kg/m^2^, TC<5.2 or ≥5.2 mmol/L, low HDL-C or normal HDL-C, with or without fatty liver disease, with or without habit of exercise, never smoking or past smoking or current smoking, and alcohol consumption (non, light, moderate, and heavy) (**Figure 4**).

### Receiver−Operating Characteristics (ROC) Curve Analysis

**Figure 5:**
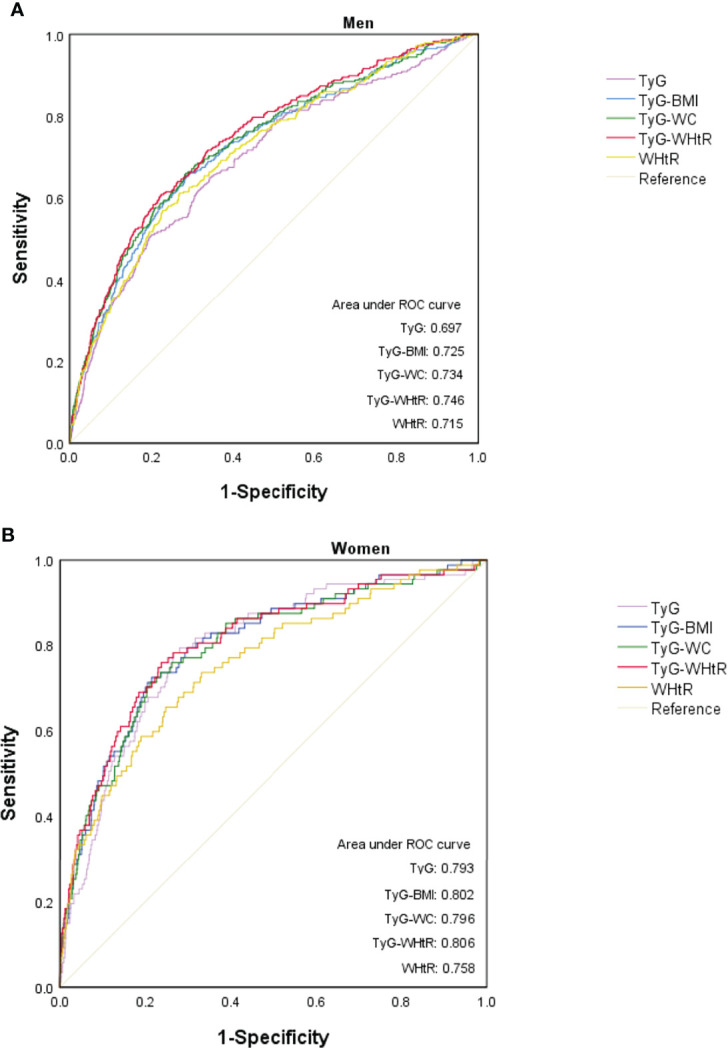
ROC curve analysis of WHtR, TyG and TyG-related parameters in men **(A)** and women **(B)**.

The diagnostic efficacy of WHtR, TyG and TyG-related parameters for diabetes according to ROC curve analysis was illustrated in **Table 3**. In men, the areas under the ROC curve (AUCs) of TyG, WHtR, TyG-WHtR, TyG-BMI and TyG-WC were 0.697 (95%CI 0.664-0.729), 0.715 (95%CI 0.684-0.746), 0.746 (95%CI 0.716-0.776), 0.725 (95%CI 0.694-0.757) and 0.734 (95%CI 0.703-0.766), respectively (All *P*<0.001). As can be seen, TyG-WHtR presented the biggest AUC compared to the other parameters and all pairwise comparisons of AUC between TyG-WHtR and other indicators were significantly different (All *P*<0.05) (**Table 4**). TyG-WHtR showed the best diagnostic value for diabetes in men, with a sensitivity of 60.8% and specificity of 77.7%. In women, the AUCs of TyG, WHtR, TyG-BMI, TyG-WHtR and TyG-WC for predicting the occurrence of diabetes were 0.793 (95%CI 0.745-0.842), 0.758 (95%CI 0.703-0.813), 0.802 (95%CI 0.752-0.851), 0.806 (95%CI 0.755-0.857) and 0.796 (95%CI 0.744-0.847), respectively (All *P*<0.001) (**Figure 5**). However, all pairwise AUC comparisons were not statistically different except TyG-WHtR vs. WHtR. The diagnostic sensitivity of TyG-WHtR was 75.9% and the specificity was 76.3% in women.

**Table 3:** ROC curves of WHtR, TyG and TyG-related parameters for predicting diabetes in men and women.

	Parameters	AUC (95%CI)	Sensitivity (%)	Specificity (%)	*P* value
Men	TyG	0.697 (0.664-0.729)	0.510	0.800	<0.001
	TyG-BMI	0.725 (0.694-0.757)	0.661	0.707	<0.001
	TyG-WC	0.734 (0.703-0.766)	0.661	0.716	<0.001
	TyG-WHtR	0.746 (0.716-0.776)	0.608	0.777	<0.001
	WHtR	0.715 (0.684-0.746)	0.612	0.732	<0.001
Women	TyG	0.793 (0.745-0.842)	0.782	0.735	<0.001
	TyG-BMI	0.802 (0.752-0.851)	0.724	0.786	<0.001
	TyG-WC	0.796 (0.744-0.847)	0.713	0.789	<0.001
	TyG-WHtR	0.806 (0.755-0.857)	0.759	0.763	<0.001
	WHtR	0.758 (0.703-0.813)	0.655	0.753	<0.001

ROC, receiver−operating characteristics; AUC: area under the curve; CI, confidence interval; TyG: triglyceride-glucose; WHtR, waist-to-height ratio; TyG-WHtR, triglyceride glucose-waist to height ratio; BMI, body mass index; WC, waist circumference.

**Table 4:** Pairwise comparison of AUC between TyG-WHtR and other parameters.

	Parameters	Differences between AUC	95% CI	*P* value
Men	TyG	0.0490	0.0260-0.0720	<0.0001
	TyG-BMI	0.0205	0.0093-0.0316	0.0003
	TyG-WC	0.0114	0.0040-0.0188	0.0026
	WHtR	0.0305	0.0133-0.0477	0.0005
Women	TyG	0.0127	-0.0209-0.0462	0.4594
	TyG-BMI	0.0044	-0.0128-0.0216	0.6172
	TyG-WC	0.0101	-0.0011-0.0213	0.0759
	WHtR	0.0480	0.0246-0.0713	0.0001

AUC, area under the curve; CI, confidence interval; TyG, triglyceride-glucose; WHtR, waist-to-height ratio; TyG-WHtR, triglyceride glucose-waist to height ratio; BMI, body mass index; WC, waist circumference.

## Discussion

This population-based retrospective longitudinal study revealed a cumulative increased risk of diabetes with ascending TyG-WHtR in Japanese population. Compared to other IR surrogates assessed by TyG index, TyG-BMI and TyG-WC, the baseline TyG-WHtR displayed the most powerful diagnostic value for incident T2DM with the highest AUC, especially in men.

The close relation between obesity and T2DM has been discussed in several previous studies (25–27). Matsuda M et al. revealed that adipose tissue was a primary source of oxidative stress, thereby contributing to obesity-associated IR and diabetes (28). Therefore, a great number of anthropometric parameters of obesity have been developed for the prediction and early detection of T2DM, such as BMI, WC and WHtR. However, which of these indicators could be regarded as a more considerable predictor of T2DM remains obscure. A systematic review and meta-analysis of 31 studies involving 123231 participants identified WHtR as having the biggest AUC for diabetes in comparison to WC and BMI (29). Similarly, study from Petermann-Rocha F et al., using data of 13044 participants from the Chilean National Health Survey, also demonstrated that WHtR was a more accurate predictor of T2DM than BMI or WC (22). In the current study, we also suggested that WHtR was the marker with a higher AUC for predicting T2DM than that of BMI and WC shown by the previous study using data from this cohort (30). The above results can be explained by the distinct roles played by BMI, WC and WHtR in assessing adiposity status. In general, BMI, a measurement based on height and weight, represents general obesity that cannot discriminate between fat and lean body mass. Additionally, the effect of adipose tissue on metabolism cannot be determined by BMI (31). Though WC stands for central obesity, it is unable to sufficiently differentiate between subcutaneous fat and visceral adipose tissue. Accumulating evidence indicated that visceral fat contributes more in developing IR and T2DM than subcutaneous fat (17). Notably, previous studies pointed to stature as an important element that made WHtR a superior predictor than WC alone. A case-cohort study demonstrated that short stature was related to IR and T2DM (32). A meta-analysis conducted in 52 cohort studies also shown that patients with shorter stature were more likely to suffer from coronary disease than taller ones (33). Hence, combining height and WC seems to be a better indicator in predicting T2DM.

After further introducing TyG index as a surrogate for IR, previous evidence has also mentioned that TyG index was prominently correlated with T2DM. A population-based retrospective cohort study from Li X et al. manifested that the risk of diabetes was 1.83 times, 3.29 times and 6.26 times, respectively higher for subjects in the increased TyG index groups (Q2, Q3, and Q4) than for those with low TyG quartile (Q1) after adjustment of potential confounders (34). Another cohort study of 5706 normal-weight participants examining the predictive value of TyG index for T2DM incidence observed that the TyG index was strongly related to future diabetes during a 6-year follow-up period (12). Likewise, in our study, the TyG index displayed the large AUC for T2DM in both men (AUC: 0.697) and women (AUC: 0.793) with a high sensitivity and specificity, indicating that TyG index may be useful for early identifying individuals who are susceptible to T2DM. This predictive power of TyG-index can potentially be explained by the close relationship between its two pivotal components (FPG and TG) and IR, which plays a vital role in the development of T2DM (35, 36). Another possible explanation may involve lipotoxicity and glucotoxicity, which contribute to the pathogenesis of T2DM (37).

Meanwhile, in this retrospective study, we considered that TyG-WHtR, the combination of TyG and WHtR, was a more robust and comprehensive predictor of diabetes incidence. Overall, every 1−unit increase of TyG-WHtR was related to a 2.714-fold higher risk of T2DM, independent of other risk factors [HR 2.714, 95% CI 1.942-3.793; *P*<0.001). Hence, it was concluded that TyG-WHtR is a recognizable and independent risk factor for T2DM. Notably, this study also highlighted the TyG-WHtR as an effective and reliable marker for detection of T2DM with the largest AUC compared to TyG, WHtR, TyG-WC and TyG-BMI. This outperformance of TyG-WHtR was particularly evident for men, which may due to the fat distribution in different gender. In line with our results, a cross-sectional study conducted in Nigerians suggested that in terms of metabolic syndrome recognition, TyG-WHtR showed the largest AUC (0.863), followed by TyG-WC (AUC: 0.858), TyG-BMI (AUC: 0.838), TyG index (AUC: 0.796), and WHtR (AUC: 0.791) in that order (38). Besides, Malek M et al. also identified that TyG-WHtR was a prominent predictor of fatty liver disease with the highest odds ratio and a largest AUC than TyG, TyG-WC and TyG-BMI (39). The superiority of TyG-WHtR may be achieved since FPG, adiposity status and TG are all taken into account, which have been well validated for their crucial roles in the development of IR and T2DM pathogenesis. However, observational study from Ke P et al. assessing the diagnostic capacity of obesity-related anthropometric parameters and TyG-derived indices for diabetes among 24215 Chinese normal-weight elderly, indicating that the relationship between TyG-WHtR and incident T2DM was weaker than for TyG alone (16). This discrepancy may result from the different age distribution of the study participants, but may be attributed to the differences in study methodology and design.

The strength of this study is its novelty, comparing the predictive capability of obesity anthropometric indices and surrogate marker of IR in predicting T2DM, which could be widely adopted in the clinic. In addition, it is a large-scale and population-based longitudinal study with broad age spectrum that guarantee robustness and dependability of the results. Several limitations also exist in the present study. Firstly, this study focused only on Japanese population. Hence, generalizability of the results to other individuals with different ethnicities and races may be limited. Secondly, the diagnosis of diabetes was based solely on FPG, or HbA1c, or patients self-reported, rather than using the two-hour oral glucose tolerance test (OGTT), which may underestimate the prevalence of diabetes to some extent. Thirdly, the TyG-WHtR was only assessed at baseline. The dynamic change during follow-up, which might be strongly associated with diabetes incidence, was not investigated in the study. Finally, the secondary analysis was limited by the disclosed data; hence, unpublished or unmeasured confounding factors in the original study could not be fully adjusted. And these residual confounders may have potential impact on the study results. Further studies are warranted to explore the effect of these factors and TyG-WHtR on the occurrence of T2DM.

## Conclusions

TyG-WHtR, comprising both TyG and WHtR, could improve the prediction and evaluation of diabetes, which is believed to outperform the traditional anthropometric indicators of obesity and other TyG-derived indicators (TyG-WC and TyG-BMI), especially in men. Since TyG-WHtR exhibits the superiority of accessibility, simplicity and efficacy, it could be widely used for early screening of diabetes in clinical settings.

## Data Availability Statement

The datasets presented in this study can be found in online repositories. The names of the repository/repositories and accession number(s) can be found in the article/supplementary material.

## Ethics Statement

The studies involving human participants were reviewed and approved by Murakami Memorial Hospital. The patients/participants provided their written informed consent to participate in this study.

## Author Contributions

WX made substantial contributions to study design, data analysis and manuscript writing. XZ organized this study, identified and dealt with the problems during the work. She was mainly responsible for the study design and data interpretation. DL made contributions to data collection, data analysis and patient data interpretation. Both JZ and HL made contributions to look up for relevant information and data interpretation. All authors participated in the manuscript writing, read and approved the final manuscript.

## Funding

This work was supported by the Dongguan Science and Technology of Social Development Program (grant numbers 20211800905442, 201950715001208, 202050715001215, 2018507150011651), the Science and Technology Planning Project of Guangdong Province (grant numbers 2020A1414010288), and the Medical Science and Technology Foundation of Guangdong Province (grant numbers C2018053, C2019097).

## Conflict of Interest

The authors declare that the research was conducted in the absence of any commercial or financial relationships that could be construed as a potential conflict of interest.

## Publisher’s Note

All claims expressed in this article are solely those of the authors and do not necessarily represent those of their affiliated organizations, or those of the publisher, the editors and the reviewers. Any product that may be evaluated in this article, or claim that may be made by its manufacturer, is not guaranteed or endorsed by the publisher.

## Abbreviations

BMI, body mass index; WC, waist circumference; WHtR, waist-to-height ratio; TyG, triglyceride-glucose; IR, insulin resistance; T2DM, type 2 diabetes mellitus; ROC, Receiver operating characteristics; AUC, area under the ROC curve; HOMA-IR, homeostasis model assessment of insulin resistance; DBP, diastolic blood pressure; SBP, systolic blood pressure; FPG, fasting plasma glucose; HbA1c, glycosylated hemoglobin A1c; HDL-C, high-density lipoprotein-cholesterol; TC, total cholesterol; ALT, alanine aminotransferase; GGT, gamma-glutamyl transferase; AST, aspartate aminotransferase; SD, standard deviation; RR, relative risk; HR, hazard ratio; CI, confidence interval; OGTT, oral glucose tolerance test.
